# Micro Cantilever Movement Detection with an Amorphous Silicon Array of Position Sensitive Detectors

**DOI:** 10.3390/s100908173

**Published:** 2010-09-01

**Authors:** Javier Contreras, Daniel Costa, Sonia Pereira, Elvira Fortunato, Rodrigo Martins, Rafal Wierzbicki, Holger Heerlein, Isabel Ferreira

**Affiliations:** 1 CENIMAT, Department of Materials Science, Faculty of Science and Technology of New University of Lisbon and CEMOP/UNINOVA, Campus da Caparica, 2928-516 Caparica, Portugal; E-Mails: dhac@fct.unl.pt (D.C.); sp@uninova.pt (S.P.); emf@fct.unl.pt (E.F.); rm@uninova.pt (R.M.); 2 NASCATEC GmbH, Ludwig-Erhard-Str. 10, 34131 Kassel, Germany; E-Mail: wierzbicki@nascatec.com (R.W.); heerlein@nascatec.com (H.H.)

**Keywords:** amorphous semiconductors, silicon, devices, systems

## Abstract

The movement of a micro cantilever was detected via a self constructed portable data acquisition prototype system which integrates a linear array of 32 1D amorphous silicon position sensitive detectors (PSD). The system was mounted on a microscope using a metal structure platform and the movement of the 30 μm wide by 400 μm long cantilever was tracked by analyzing the signals acquired by the 32 sensor array electronic readout system and the relevant data algorithm. The obtained results show a linear behavior of the photocurrent relating X and Y movement, with a non-linearity of about 3%, a spatial resolution of less than 2 μm along the lateral dimension of the sensor as well as of less than 3 μm along the perpendicular dimension of the sensor, when detecting just the micro-cantilever, and a spatial resolution of less than 1 μm when detecting the holding structure.

## 1. Introduction

Interest in optical microscopy applications for micron and submicron research has grown enormously in a wide range of disciplines during the last ten years [1–5]. Thereby, the need to perform tracking and record movements or dimensions of objects under the microscope is clear in many scientific applications. Various software applications have been developed to track the movement of microstructures under the microscope. Usually the imaging system associated to these operations is composed by a video camera, a frame grabber card and a personal computer [6]. Nowadays, the position/movement of micro objects can be filmed by a high quality camera with more than 50 images per second. Every single image of the CCD camera is then analyzed in real-time by image processing algorithms in order to extract and obtain the desired information. However, this whole process is too slow for some applications. Unlike detectors which are formed of discrete elements such as for example, CCDs, PSDs provide continuous position information offering a high speed response and a superb position resolution, plus being able to detect simultaneously the intensity and the position of the centre of gravity of a light spot. For example, to date, fast feedback techniques have relied on quadrant photodiode detectors [7], which measure the position of the image of an optically trapped object at rates up to 10 kHz. Position sensitive detectors are simple photodiodes which are able to measure the position of a light spot projected on their surface. These optical position sensors consist of highly resistive semiconductor substrates on which a uniform resistive layer is formed. There is an electrode or contact at each side of the resistive layer in order to acquire the intensity signal which is then be used to calculate the relevant position signal. Photocurrent is generated via a semiconductor junction due to the photovoltaic effect as described by Wallmark [8]. Similarly to what happens with Charge Coupled Device Detectors (CCD) [9] the data recorded are computer processed, but now with a major advantage: the detection can work in a continuous mode, not limited to the 50 or 100 frames per second rate. Besides that, for 2D resolution, the number of strips will supply lateral digital resolution, while analogue information is supplied by each sensor strip along its length. For a 32-element linear array position sensitive detector, the lateral resolution is only of 5 bits, but the acquisition rate of a surface/contour is larger than 5 × 10^3^ measurements per second [10]. This value is one order of magnitude larger than for most CCD cameras, while the bit resolution could be improved by increasing the number of array elements (256, for 8 bits resolution). This makes these sensors quite suitable for supplying information on position and angle sensing; distortion and vibration measurements; lens reflection and refraction measurements, laser displacement sensing; optical switches and other applications. That is, PSDs are ideal for any application requiring low levels of signal processing power or high speeds in comparison to existing standard video frame rates. The use of amorphous silicon pin structures as PSDs has been already reported [11,12]. Since then, the use of such structures as PSDs has been increasing. Fabrication details, architecture and working principle of these 1D, 2D and 3D PSD detectors were already described in previous papers [12–15], presenting good characteristics for position detection of a direct incident laser beam with a linear resolution of 1 μm [16]. These types of sensors have also been used for the detection of micro objects, based on reflecting light coming from those objects [17] and no further work relating these sensors towards the detection of small objects has been reported.

The following work proposes the use of these sensors and relevant system for detecting micro object movement and dimensions even at high speeds. This paper shows how a portable prototype system mounted on the microscope and integrating an array of 32 position sensitive line sensors has been used to detect the movement and dimensions of a commercial micro cantilever provided by NASCATEC.

The results have shown good detection and a linearity of about 97% along the lateral dimension of the sensor. The movement of the micro cantilever can also be properly detected with a resolution of better than 3 μm (1 μm corresponds to approx. 0.305 nA). The offset noise level present in reduced ambient light conditions varies from a minimum value of 0.278 nA to approximately 0.417 nA, and never exceeds a maximum threshold value of 0.556 nA.

## 2. Working Principles of a Position Sensitive Detector Array

**Figure 1.**
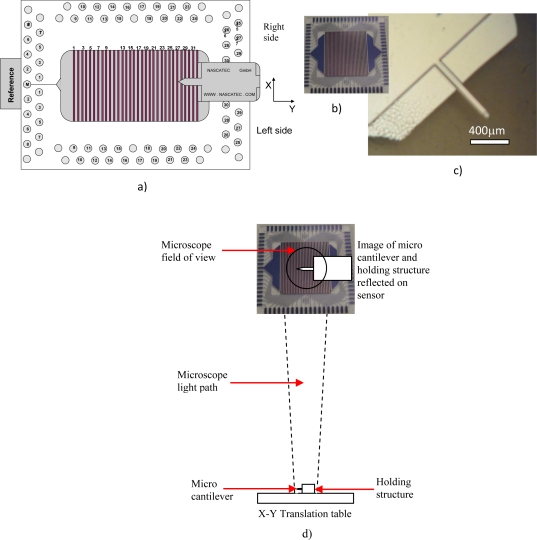
**(a)** Sketch of the 32 PSD sensor with appropriate line numbering **(b)** Photograph of the 32 PSD sensor **(c)** Photograph of the micro cantilever and its holding structure **(d)** Sketch of the light path, sensor, micro cantilever and reflected image setup.

The operation principle of a PSD array is that an image line projected in the array (see Figure 1) induces photocurrents (*I_ph1i_* and *I_ph2i_*) or lateral photo voltages (*ΔV* = *V_1i_* − *V_2i_*) in the illuminated elements, where 1 and 2 refers to two terminals of the same strip of the array and i refers to the strip number, that varies from 1 to 32, for the present case. All elements are then scanned with a certain velocity, able to determine the position of the image line. This depends on the angle of incidence *ϕ* that the laser line makes with the surface to be inspected that determines the relation between the movement of the light line (*d_d_*) and the object movement (*d_0_*) [10]:

(1) $$d_{0}(\text{max})\geq \frac{d_{d}(\text{max})}{\text{cos}\varphi }$$

The photocurrent of each element Δ*I_ph_* has an uncertainty related to the noise (*n*). The measured position is given by [11]:

(2) $$P(y_{n})=\frac{\left(I_{ph_{1i}}\pm n_{1i})-(I_{ph_{2i}}\pm n_{2i}\right)}{\left(I_{ph_{1i}}\pm n_{1i})+(I_{ph_{2i}}\pm n_{2i}\right)}\cdot \frac{L}{2}\Rightarrow P_{\text{max}}(y_{n})=\frac{I_{ph_{1i}}-I_{ph_{2i}}}{I_{ph_{1i}}+I_{ph_{2i}}-2n}\cdot \frac{L}{2},\text{for }n_{\mathrm{1i}}=n_{\mathrm{2i}}=n$$

where *n_1i_* and *n_2i_* are the absolute values of noise detected at each of the element terminals and *L* is the length of each line. Thus, the position of an image line projected in the plane *z−y* is determined by *P(y_n_)* obtained by the 32 stripes and related to the currents detected by both shift registers (*SR*) connected to the terminals of the PSD array.

The detection threshold limit will depend on the signal to noise ratio (*S/N*), which is given by $S/N=\frac{I_{ph_{1}}+I_{ph_{2}}}{2n}$. Thus, the positional resolution (*dP*) depends on the active length of each sensing element and on (*S/N*): $dP\approx \frac{L}{2S/N}$ [11].

## 3. Experimental Setup

**Figure 2.**
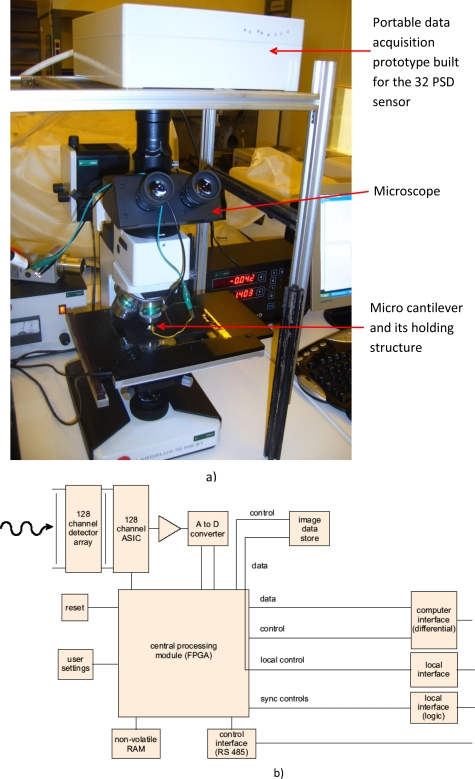
**(a)** Photograph of the experimental setup including the built data acquisition prototype, the microscope and the micro cantilever together with its holding structure **(b)** Schematic diagram of the existing commercially available electronics module.

The experimental setup used to test the 3D PSD, whose sketch and photograph are shown in Figures 1(a,b) and 2(a), respectively, is a common microscope (Leitz Laborlux 12 ME ST) working in the reflective mode. The sensor was fabricated as described elsewhere [15] and was placed on top of the vertical objective where a digital camera is usually connected.

This way, the light arriving to the sensor is the reflected light from the object that is then being focused onto the 3D sensor. The active area of the sensor is 0.7 cm × 1.7 cm but, after focusing, the illuminated area of the sensor is around 1 cm in diameter. Thus, the whole measurements are related to an area of approximately 0.5 cm^2^. The intensity of the light (lamp of the microscope) focused on the sensor varies from a background light (using a black surface) level of about 9 μW/cm^2^, to a maximum light level reflected by the object of about 700 μW/cm^2^. The object consists of a micro cantilever and its holding structure as shown in the photograph of Figure 1(c). The dimensions of the cantilever are approximately 400 μm in length by 30 μm in width, and the sensor is able to detect the movement not only of the holding structure, but also of the micro cantilever [see Figure 1(c)]. The experimental setup is shown on the photograph of Figure 2(a). The procedure used for the measurements was the following: the sensor was fixed on top of the focusing lenses and the object was placed on the X-Y moving table and then focused by using the microscope embedded magnification lenses. The photocurrent of the 32 1D sensors was also measured at background reflected light level (without the object appearing within the image scope), giving the previously mentioned offset noise signal level response. The complete set of measurements was always recorded when the cantilever was appearing on the ocular of the microscope and when it was moving in the X and Y direction, respectively, in controlled steps of 5 μm.

In all of these experiments, the micro object is a cantilever. The whole set of results have been taken with and without using a focusing lens placed before the sensor. However, when the micro cantilever was immersed inside a liquid, a focusing lens was always present and the liquid used was propanol. Nowadays, there are many scenarios in which micro objects are residing inside a liquid. Due to this, it was interesting and useful to check if a movement signal is being detected when the medium is changed from air to liquid (propanol). The refractive index changes from air to liquid and so it is expected that the signal might not detected as well as when the medium is air.

## 4. Electronics Module

The system is comprised of a commercially available electronics module suitable for photodiode data acquisition operations and by another adapter module which allows for removal and replacement of the 32 PSD based sensor whenever needed. A schematic of the system electronics module responsible for the data acquisition and control is shown in Figure 2(b). A 32 channel detector array developed at CEMOP/UNINOVA [15] is connected via an adaptor printed circuit board to a specially manufactured integrated circuit (ASIC), which amplifies and subsequently multiplexes the input signals. An A/D converter then converts the signals from analogue to digital and passes the data to a field programmable gate array (central processing module––FPGA). This module controls the operation of the system and sends the processed data to the PC and communicates with the other modules and devices via the control lines shown in Figure 2(b) sending the data through the data lines. Other modules are used, for example, to save user settings or image information. The system can acquire a single line of data in a minimum time of 10 μs, performs simultaneous data acquisition and read-out, offers a wide dynamic range, delivers 16 bit output and has a high speed SCSI-USB link to the PC.

## 5. Results and Discussion

The results discussed in this paper are related to the signal measured by a specially developed prototype system with 0 bias voltage applied to the sensor. That is, the sensor works in the photovoltaic mode [14]. This mode is suitable for low light level and low frequency applications and it allows simplicity in system design and development. Response speed and linearity could improve via the application of a reverse bias, however, dark and noise currents as well as response variations due to temperature are likely to increase.

When the micro object is moving in the direction perpendicular to the sensor lines, the 1D/3D PSD detector line numbering adopted is that one depicted in Figure 1(a), where the micro object movement started at the highest number. However, when the micro object was moving in the direction parallel to the sensor lines, movement was taking place along a few sensor lines usually in the middle of the sensor (e.g., line number 17, 18).

**Figure 3.**
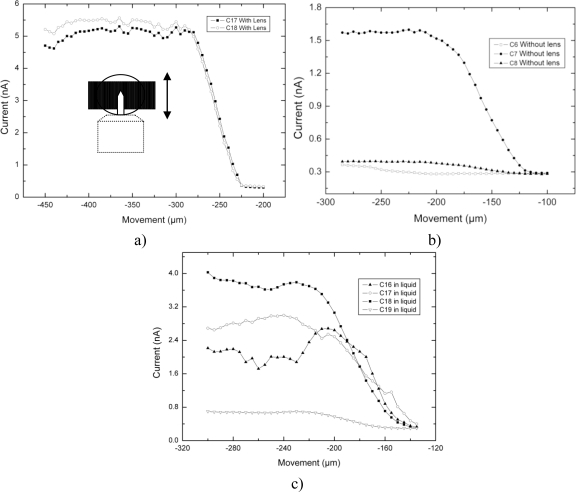
**(a)** Sketch of the micro cantilever entering parallel to the sensor lines. It enters approximately at the centre of the sensor at detector 17 or 18 **(b)** Sketch of results according to when no focusing lens is used **(c)** Sketch of results for when the micro cantilever is immersed inside a liquid.

All three different experimental results presented in Figures 3(a–c) show the response of the sensor as the micro cantilever enters in parallel to its line sensor. According to the numbering scheme in Figure 1(a), the micro cantilever enters the sensor at line detector numbers 17, 18 for the case of Figures 3(a,c) or at line detector number 7 for the case of Figure 3(b). Movement detection happens as it moves in parallel to them. In Figure 3(a), a focusing lens is used and channels 17 and 18 (C17, C18) detect, in Figure 3(b), no lens is used and channel 7 (C7) detects and in Figure 3(c), the micro cantilever is immersed in liquid and channels 17, 18 and 19 (C17, C18, C19) detect. The latter signal responses are a measurement of current (nA) *versus* position movement (μm). The instability presented on some signals is believed to be related to the flickering caused by the microscope lamp.

Liquid made it difficult to maintain the focus. In Figure 3(c), the relevant three line sensors or channels (C17, C18, C19), obtain a signal response more than double that given by the noise level. The reason why there is instability coming from the response of at least one detector is because the slightest movement of the liquid was causing at some instances the loss of image focus on the sensor.

**Figure 4.**
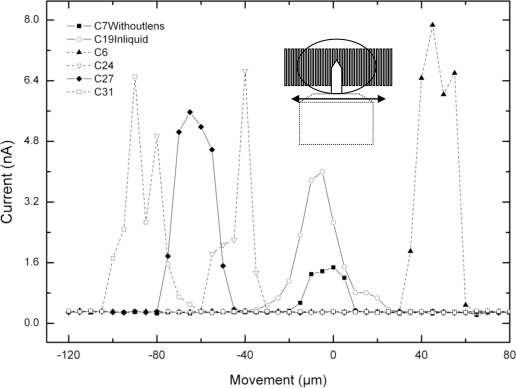
Sketch of the micro cantilever moving sideways, after having entered parallel to the sensor lines as in Figure 3.

The set of results shown in Figure 4 derive from the signal related to the movement detection coming from just the micro cantilever, which is being reflected on the viewing area of the microscope and do not derive from the signal linked to the movement detection coming from its holding structure. Now the micro cantilever was moved from side to side perpendicular to the detector lines. All channels except channel 7 (C7) show the response when the focusing lens is present and among these, channel 19 shows the response when the micro cantilever was moving inside a liquid. Channel 7 (C7) represents the behavior when no focusing lens is present. The viewing area of the microscope is projected around channel 7 when no focusing lens is present and around channels 17 or 18 when a focusing lens is present. An interesting observation from the results of Figure 4 is that all channels are detecting for approximately 30 μm and this happens to be the same as the width of the micro cantilever.

**Figure 5.**
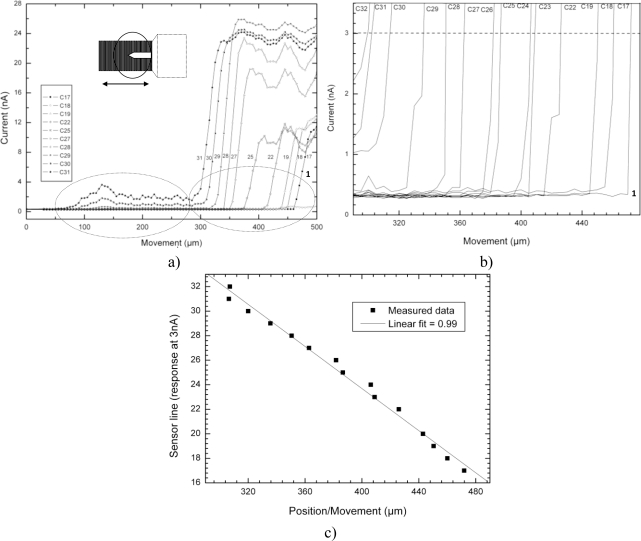
**(a)** Sketch of the micro cantilever and its holding structure entering perpendicular to the sensor lines **(b)** Sketch of the response of each detector at the 3 nA threshold level, when just the holding structure of the cantilever is present **(c)** Sketch of the measured data at each detector for the 3 nA threshold level and its related linear fit.

The set of results in Figure 5(a), show the response of the sensor as the micro cantilever enters perpendicular to it and therefore perpendicular to its channels too. These results also show the difference in terms of current (nA), between the signal linked to the movement detection coming from the micro cantilever and the signal related to the movement detection coming from its holding structure. As previously stated, the micro object was moving in the direction perpendicular to the sensor lines and the line numbering adopted is that one indicated in Figure 1(a), where movement started at the highest number. For these particular results, the movement of the micro cantilever and its holding structure were both scanned at steps of 5 μm.

In Figure 5(a), the signal related to the movement detection coming from the micro cantilever extends from 100 μm to about 300 μm. This indicates that only the first 200 μm from the length of the micro cantilever are detected, as opposed to 400 μm, which is the real length of the micro cantilever. The remaining 200 μm of micro cantilever are believed to be included (and masked) in the signal detecting the holding structure which starts after 300 μm. It is at that point that the holding structure starts to enter the field of view and the signal response boosts rapidly stabilizing at that level. The difference between the light reflected by the micro cantilever and the holding structure is therefore clearly seen. As shown in Figure 1(c), the holding structure has a much bigger cross sectional area than the micro cantilever and therefore reflects much more light. The instability present on some signals is believed to be related to the flickering caused by the microscope lamp.

Figure 5(b) shows the response (as a function of position/movement) obtained by all line sensors just for the signal related to the holding structure (not the signal related to the micro cantilever) at the 3 nA line, which cuts across in order to help determining linearity of the sensor in this particular situation, which is next sketched in Figure 5(c).

Figure 5(c) presents the measured data by all sensors lines when they are detecting exactly at the 3 nA threshold. As the holding structure moves along (position in μm), the line sensors are detecting sequentially and in the right order as it should be. The linearity of the sensor is thus represented by the linear fit and the correlation coefficient is 0.99 (99%).

**Figure 6.**
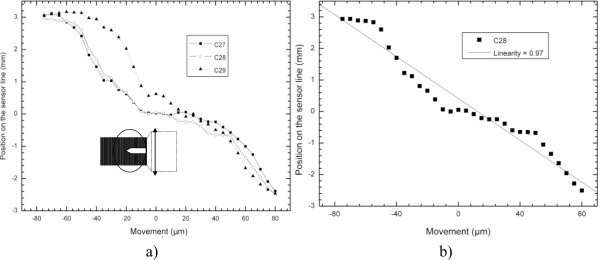
**(a)** Sketch of the micro cantilever moving sideways, after having entered perpendicular to the sensor lines as in Figure 5. **(b)** Sketch of the best channel response from Figure 6(a) and its related calculated linearity.

The set of measurements shown in Figure 6(a) derive from the signal related to the movement detection coming from just the micro cantilever, and do not derive from the signal linked to the movement detection coming from its holding structure. Once the micro cantilever is in the viewing area of the microscope, its movement is recorded as it is moved from one side to the other side of the sensor lines, in the indicated direction of movement. This time, the micro cantilever is moved along the lateral dimension of the detector lines and a focusing lens was used. The position signal response coming from several line sensors (C27, C28 and C29), clearly shows a difference between moving from left to right of the sensor. Each detector line measures 7mm and therefore the range extends from −3.5 mm to +3.5 mm. The reason why some channels perform better than others is because of the internal material characteristics of each detector resultant from the fabrication.

Figure 6(b) shows only channel 28 (C28) from Figure 6(a) because it is the sensor line that responds best to position detection. The sketched black line represents the linearity of this particular sensor, in this particular situation, in which the linear correlation coefficient is 0.97 (97%).

## 6. Conclusions

The overall results obtained show the possibility of using a linear array of 32 1D line sensors based on the amorphous silicon technology to detect the movement, dimensions and 2D position of micrometer objects like a cantilever. It can be concluded that placing a focusing lens before the sensor is preferred since a higher overall channel intensity signal is obtained. From the set of experimental results obtained in this work, it can also be concluded that the best way to detect the movement of the micro cantilever is when it enters in the field of view parallel to the sensor lines. There is also a logical relationship between the cross sectional area of the object appearing in the field of view and the level of signal acquired. Of course, the greater the area of the object, the higher the light reflected.

As previously stated, in terms of dimensions, Figure 4 shows all channels detecting for approximately 30 μm and this happens to be the same as the width of the micro cantilever. In Figure 5(a), only the first 200 μm from the length of the micro cantilever are detected, as opposed to 400 μm, which is the real length of the micro cantilever. The remaining 200 μm of micro cantilever are believed to be included (and masked) in the signal detecting the holding structure (which starts after 300 μm in Figure 5(a).

Concerning positioning, X and Y positions (2D) can be determined based on the information and/or the shape of the signal responses obtained. Finally, X and Y movement can be detected with a resolution which will basically depend on the step movement of the table were the object is placed, the precision of the electronic readout system, and the optics used. That is, it depends on the minimum values of photocurrent that the electronic components can detect.

This kind of setup offers the possibility to detect if a micro object is moving, what are its dimensions and what is its position in two dimensions, even at high speeds. In microscopy applications, a micro object could be moving on the translation table of the microscope and reflecting the microscope light incident on it as an image onto the 32 linear array of 1D line sensors. Movement signals would be detected as it starts to appear perpendicular to the sensor and when it has already passed by and these will define one dimension (e.g., length) and will also determine the Y position of the reflected micro object on the sensor. The other dimension (width) and the X position would be detected when the object moves parallel to the sensor along the line sensors. On top of that it would be possible to detect those movement signals automatically if an object moves below the microscope by being able to trigger some kind of warning if movement occurred.
